# HuR and Ago2 Bind the Internal Ribosome Entry Site of Enterovirus 71 and Promote Virus Translation and Replication

**DOI:** 10.1371/journal.pone.0140291

**Published:** 2015-10-09

**Authors:** Jing-Yi Lin, Gary Brewer, Mei-Ling Li

**Affiliations:** 1 School of Medical Laboratory Science and Biotechnology, College of Medical Science and Technology, Taipei Medical University, Taipei, Taiwan; 2 Department of Biochemistry & Molecular Biology, Rutgers Robert Wood Johnson Medical School, Piscataway, New Jersey, United States of America; University of British Columbia, CANADA

## Abstract

EV71 (enterovirus 71) RNA contains an internal ribosomal entry site (IRES) that directs cap-independent initiation of translation. IRES-dependent translation requires the host’s translation initiation factors and IRES-associated trans-acting factors (ITAFs). We reported recently that mRNA decay factor AUF1 is a negative-acting ITAF that binds IRES stem-loop II. We also reported that the small RNA-processing enzyme Dicer produces at least four small RNAs (vsRNAs) from the EV71 IRES. One of these, vsRNA1, derived from IRES stem-loop II, reduces IRES activity and virus replication. Since its mechanism of action is unknown, we hypothesized that it might control association of ITAFs with the IRES. Here, we identified the mRNA stability factor HuR and the RISC subunit Argonaute 2 (Ago2) as two ITAFs that bind stem-loop II. In contrast to AUF1, HuR and Ago2 promote EV71 IRES activity and virus replication. In vitro RNA-binding assays revealed that vsRNA1 can alter association of Ago2, HuR, and AUF1 with stem-loop II. This presents a possible mechanism by which vsRNA1 could control viral translation and replication.

## Introduction

Enterovirus 71 (EV71), a member of the family Picornaviridae, poses a persistent global public health problem. EV71 infections usually cause hand-foot-and-mouth disease (HFMD) or herpangina, yet EV71 has also been implicated as the etiological agent in several large-scale outbreaks of severe neurological disorders in children worldwide [1]. In recent years, an increase of EV71 endemic activity has been noted throughout the Asia-Pacific region [2–6]. Severe neurological complications, including brainstem encephalitis, meningitis, poliomyelitis-like paralysis, and death have occurred in theses endemics. In 1998, an EV71 endemic occurred in Taiwan, with the virus infecting over 120,000 people and killing 78 children. Many other EV71 endemics of smaller scale have also occurred after 1998 in Taiwan [2].

EV71 is a positive-stranded RNA virus [7]. The viral RNA has a small protein called VPg covalently attached to its 5’-end; viral RNA is polyadenylated at its 3’-terminus. The genomic RNA is around 7,500 nucleotides long. The 5’-untranslated region (5’ UTR) is 745 nucleotides long and highly structured, and contains both a cloverleaf-like structure for viral RNA synthesis and an internal ribosomal entry site (IRES) for cap-independent translation. IRES-dependent translation depends on host translation initiation factors and IRES-associated trans-acting factors (ITAFs). The 40S ribosomal subunit recognizes a sequence, RNA structure, or ribonucleoprotein complex within the IRES, and initiation occurs at the authentic start codon [7]. Many cellular factors associate with the EV71 5’ UTR and participate in viral RNA replication and/or viral IRES activity. These factors include poly(rC)-binding protein 1 (PCBP1), PCBP2, polypyrimidine tract binding protein (PTB), heterogeneous nuclear ribonucleoprotein A1/A2 (hnRNP A1/A2), hnRNP K, La, Upstream of NRAS (Unr), far upstream element binding protein 1 (FBP1), FBP2/KSRP (KH-type splicing regulatory protein), Sam68 (68-kDa Src-associated protein in mitosis), AU-rich element binding factor 1 (AUF1), and virus-derived small RNA 1 (vsRNA1) [8–17].

Stem-loop II (SL-II) within the EV71 5’ UTR is essential for EV71 viral translation. Our previous studies showed that hnRNP A1 and AUF1 associate with this region [12,15]. While hnRNP A1 enhances IRES-dependent translation, AUF1 inhibits translation. However, EV71-infected cells generate at least four small, virus-derived RNAs, vsRNA1-4, by cleavage of the 5’UTR by Dicer [16]. vsRNA1, which originates from IRES SL-II, reduces IRES-dependent translation by unknown mechanisms. We hypothesized that vsRNA1 might control association of ITAFs with the IRES. We therefore tested for additional ITAFs in the present work. We found that mRNA stabilizing protein HuR and Ago2, a subunit of the miRNA-induced silencing complex (RISC), associate with SL-II. Ago2 and HuR knockdown decreased IRES activity, virus protein level, and virus titer, indicating that Ago2 and HuR are positive regulators of EV71 replication. vsRNA1 promoted association of AUF1, Ago2, and HuR with SL-II. Thus, vsRNA1 might control EV71 translation and replication via its impact on ITAF-IRES interactions.

## Materials and Methods

### Cells and virus

SF268 (human glioblastoma) [18], RD (human embryonal rhabdomyosarcoma) [19] and Vero (African green monkey kidney) [20] cells were cultured as described [15]. EV71 (TW/2231/98) was propagated in RD cells. Cells were infected with EV71 at the indicated multiplicity of infection (MOI) and then incubated at 37°C for 1 h for adsorption. Unbound virus was removed, and cells were refed fresh medium. Media from infected cultures were harvested at the indicated times, and virus titers were determined by plaque assay on Vero cells.

### RNP-immunoprecipitation

Immunoprecipitations of endogenous protein-RNA complexes were used to assess association of Ago2 and HuR with EV71 RNA in infected cells as described [15]. RNAs were then analyzed by Northern blot as described [15].

### Plasmid construction and in vitro transcription

The plasmid pT7-EV71 5’ UTR was constructed as described [15]. RNAs were synthesized with the MEGAscript T7 kit (Life Technologies) according to the manufacturer’s protocol. Biotinylated RNA was synthesized in a 20 μl MEGAscript transcription reaction mixture by addition of 1.25 μl of 20 mM biotinylated UTP, Bio-16-UTP (Roche). Synthesized RNAs were purified with the RNeasy Mini Kit (Qiagen) and analyzed on 1% agarose gels.

The pRHF, biscistronic luciferase reporter plasmid was described previously [11]. The plasmid contains a T7 promoter, downstream of the CMV promoter to permit synthesis of RNA. RLuc-EV71-5’UTR-FLuc RNA was synthesized as described [15].

### Pull-down of protein-biotinylated RNA complexes with streptavidin beads

SF268 cells were grown in RPMI medium supplemented with 10% fetal bovine serum (Mediatech). Upon confluence, cell extracts were prepared, and pull-down assays of complexes between cellular proteins and biotinylated RNA were performed as described [15]. Proteins bound to biotinylated RNA were fractionated by 10% SDS-PAGE, and specific proteins were detected by Western blotting analyses as described below. In some experiments, synthetic, mimic vsRNA1 or a synthetic, control RNA that contains scrambled vsRNA1 sequence was added to reactions prior to pull-down of biotinylated RNA [16]. These RNAs were synthesized by GeneDireX (Taiwan).

### Knockdown of AUF1, Ago2 and HuR

Two micrograms of plasmid expressing control short hairpin (sh)RNA or target shRNA were mixed in 100 μl serum-free MEM and combined with 10 μl SuperFect reagent (Qiagen); this mixture was incubated at room temperature for 10 min before addition to cell cultures. Knockdown efficiency was monitored by Western blotting. Short hairpin plasmids: Control, pSilencer-U6-hygro/shCTRL; shAUF1, pSilencer-U6-hygro/shAUF1 [21]; shAgo2-1 and shAgo2-2 (kindly provided by Dr Shobha Vasudevan) [22]; shHuR, pcU6K/shHuR [23].

### Western blot analysis

Western blot was carried out as described [15]. Primary antibodies were used at the following dilutions or concentrations: anti-AUF1 rabbit polyclonal, 1:15,000; anti-Ago2 rabbit polyclonal (Abcam), 1:200; anti-HuR mouse monoclonal (Santa Cruz), 1:200; anti-hnRNP A1 mouse monoclonal (Abcam), 1:200; anti-hnRNP A2 mouse monoclonal (Abcam), 1:200; anti-3C mouse polyclonal [24], 1:750; anti-β actin rabbit polyclonal (Abcam), 1:5,000. For sequential detection of different proteins, antibodies were removed from membranes by washing them with OneMinute stripping buffer (GM Biosciences).

### Determination of viral RNA replication

SF268 cells were infected with EV71 at an MOI = 2. Cells were harvested at various time points, and total RNA was extracted from cells with an RNeasy minikit (Qiagen). Viral RNA was determined by real time RT-PCR as described [15].

### Determination of EV71 IRES activity

SF268 cells were seeded in 12-well plates in antibiotic-free RPMI and transfected with RLuc-EV71-5’UTR-FLuc RNA as described [15]. IRES activity was determined two days after transfection by measuring Renilla luciferase (RLuc) and Firefly luciferase (FLuc) activities with a dual-luciferase reporter assay system (Promega) according to the manufacturer’s instructions.

## Results

### Ago2 and HuR associate with EV71 RNA in infected cells

**Fig 1:**
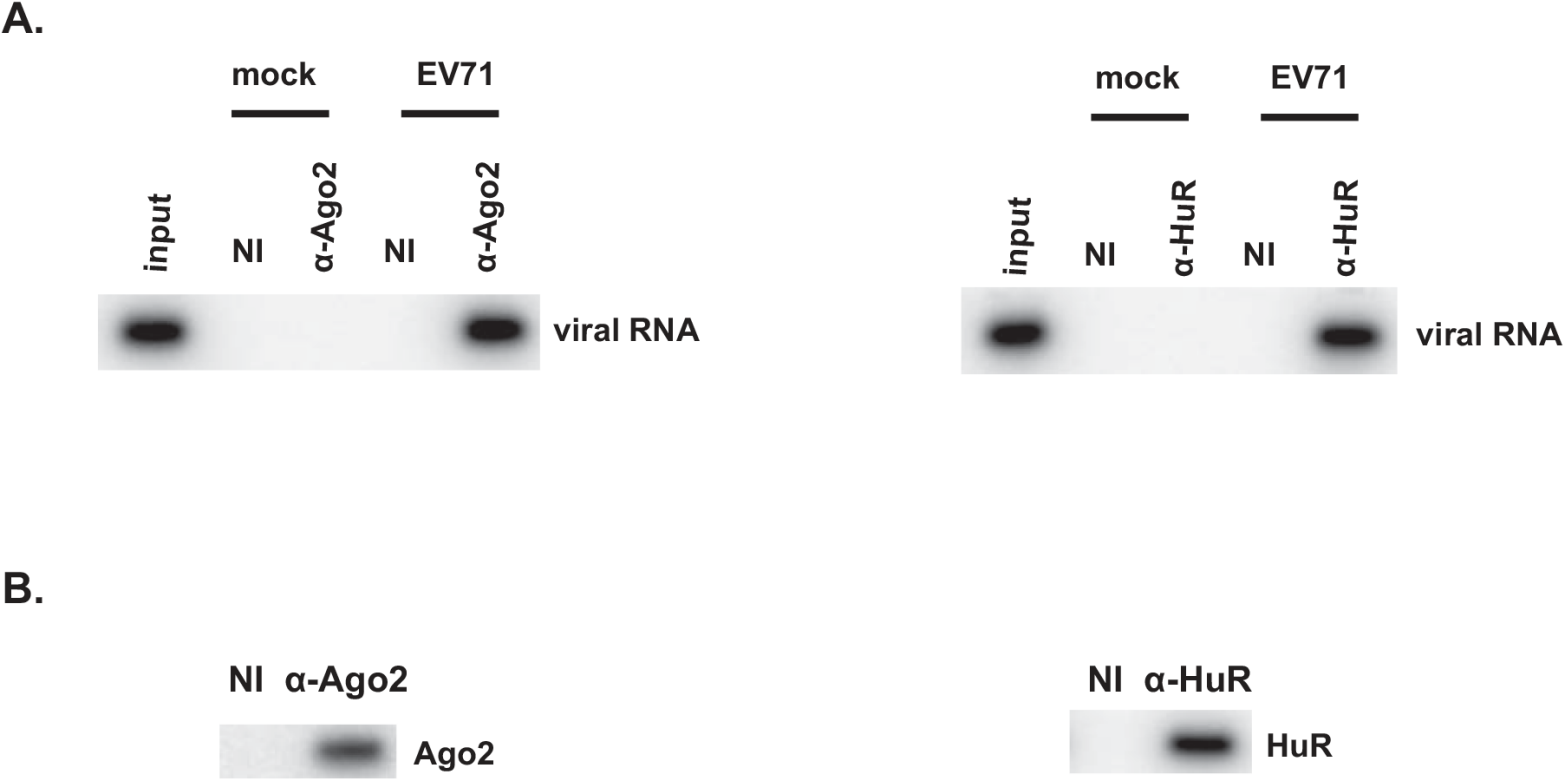
Ago2 and HuR associate with the EV71 RNA in infected cells. **(A)** Lysates of mock- or EV71-infected SF268 cells were prepared and analyed by ribonucleoprotein immunoprecipitation (RIP) with non-immune serum (N.I.) or Ago2 or HuR anti-sera. EV71 RNA was analyzed by Northern blotting. **(B)** Portions of immunoprecipitated materials were analyzed by Western blot to verify anti-Ago2 or anti-HuR–dependent recovery of Ago2 or HuR, respectively.

To assess whether Ago2 and HuR associate with EV71 viral RNA in infected cells, RNP immunoprecipitation and Northern blotting were performed. SF268 cells were infected with EV71 at an MOI = 40 and cell lysates were prepared 8 h post-infection. RNA-protein complexes were immunoprecipitated with non-immune rabbit serum or antiserum specific to Ago2 or HuR. RNA was isolated from the immune precipitates and analyzed by Northern blotting with a radiolabeled probe specific to EV71 RNA. Immunoprecipitation with Ago2 or HuR antibodies co-precipitated EV71 RNA while non-immune serum did not (Fig 1A). No RNA of similar size from mock-infected cells co-precipitated with Ago2 or HuR indicating specificity of the probe for EV71 RNA. Western blot analyses of immunoprecipitated materials confirmed specific precipitations of Ago2 and HuR by their respective antibodies (Fig 1B). Taken together, these results indicate that Ago2 and HuR associate with EV71 RNA in infected cells.

### Ago2 and HuR interact with SL-II of the EV71 5’UTR

**Fig 2:**
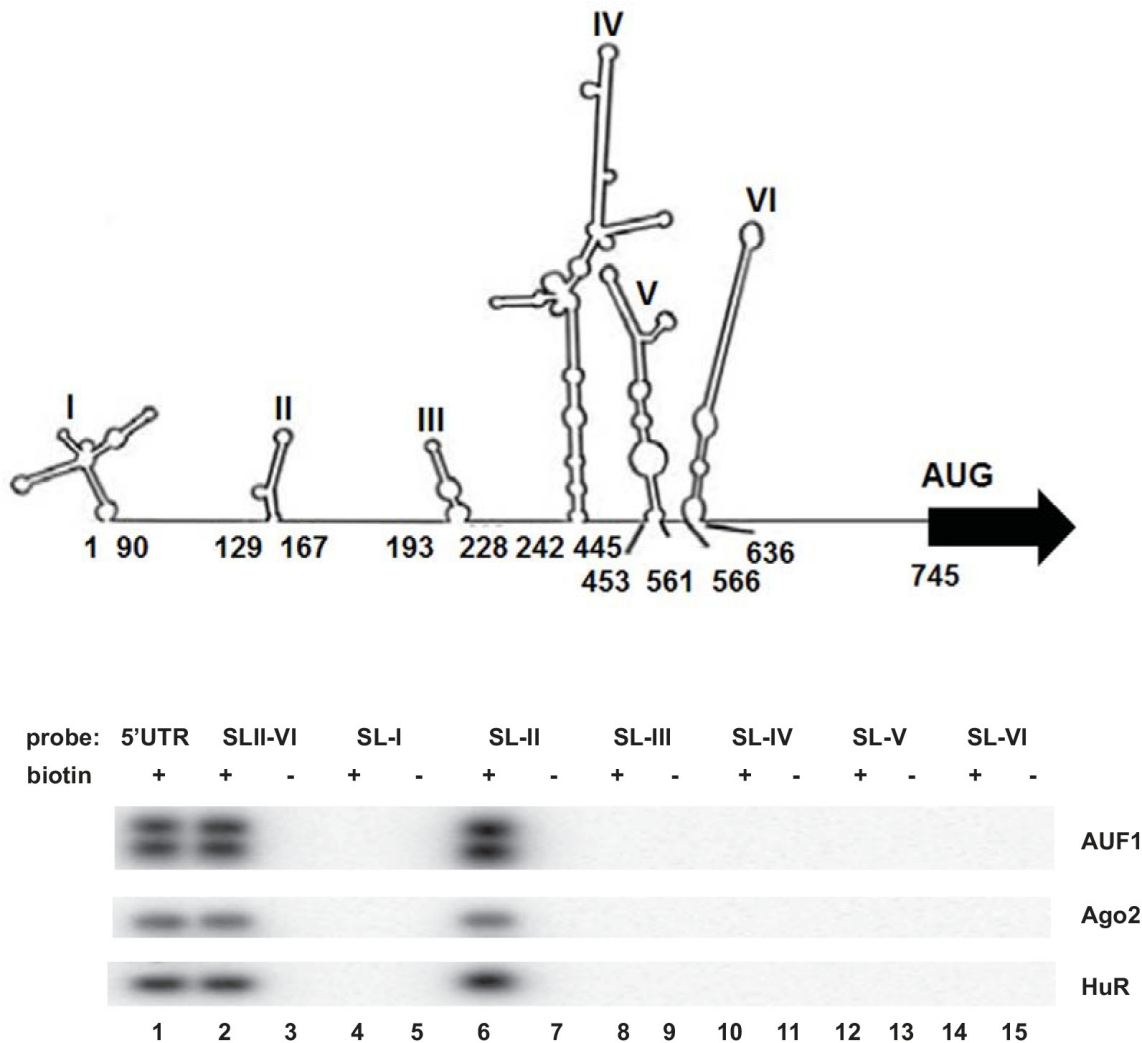
Ago2 and HuR associate with SL-II within the EV71 5’UTR. **(A)** Secondary structures within the 5’UTR were predicted by MFold as depicted earlier [15]. Numbers below each stem-loop indicate 5’UTR nucleotides corresponding to each stem-loop. The RNA substrates used for protein-RNA pull-down experiments also contained the flanking region immediately 5’ to a given stem-loop. **(B)** Identification of Ago2 and HuR interaction sites within the EV71 5’UTR. RNA-protein pull-down experiments were performed to examine the interaction between Ago2, HuR, and AUF1 with the EV71 5’UTR segments. Biotinylated RNAs corresponding to the indicated stem-loops and immediate 5’ flanking region were synthesized; control RNAs lacked biotin. Recovered proteins in the pull-downs were detected by Western blotting analyses. As expected, AUF1 binds SL-II [15].

The EV71 5’ UTR contains a 5’ cloverleaf-like structure important for viral RNA synthesis and an internal ribosomal entry site (IRES) that is critical for viral mRNA translation. To determine the region(s) of the 5’UTR with which Ago2 and HuR interact, different stem-loops of the EV71 5’UTR were synthesized in vitro with biotin-UTP. These regions are shown in Fig 2A. These RNAs were mixed with cell extracts and protein-biotinylated RNA complexes were recovered with streptavidin-sepharose; Western blotting of recovered materials was carried out to detect Ago2 and HuR proteins. Ago2 and HuR associated with SL-II of the EV71 5’ UTR (Fig 2B). As expected, no pull-downs of Ago 2 and HuR were observed when unlabeled counterparts of the EV71 5’UTR were used. The blot was stripped and re-probed with anti-AUF1 as a positive control. Our previous study showed that AUF1 associates with SL-II [15]. These experiments indicate that, similar to AUF1, Ago2 and HuR interact with the IRES SL-II.

### Effects of Ago2 and HuR on EV71 IRES activity and viral replication

**Fig 3:**
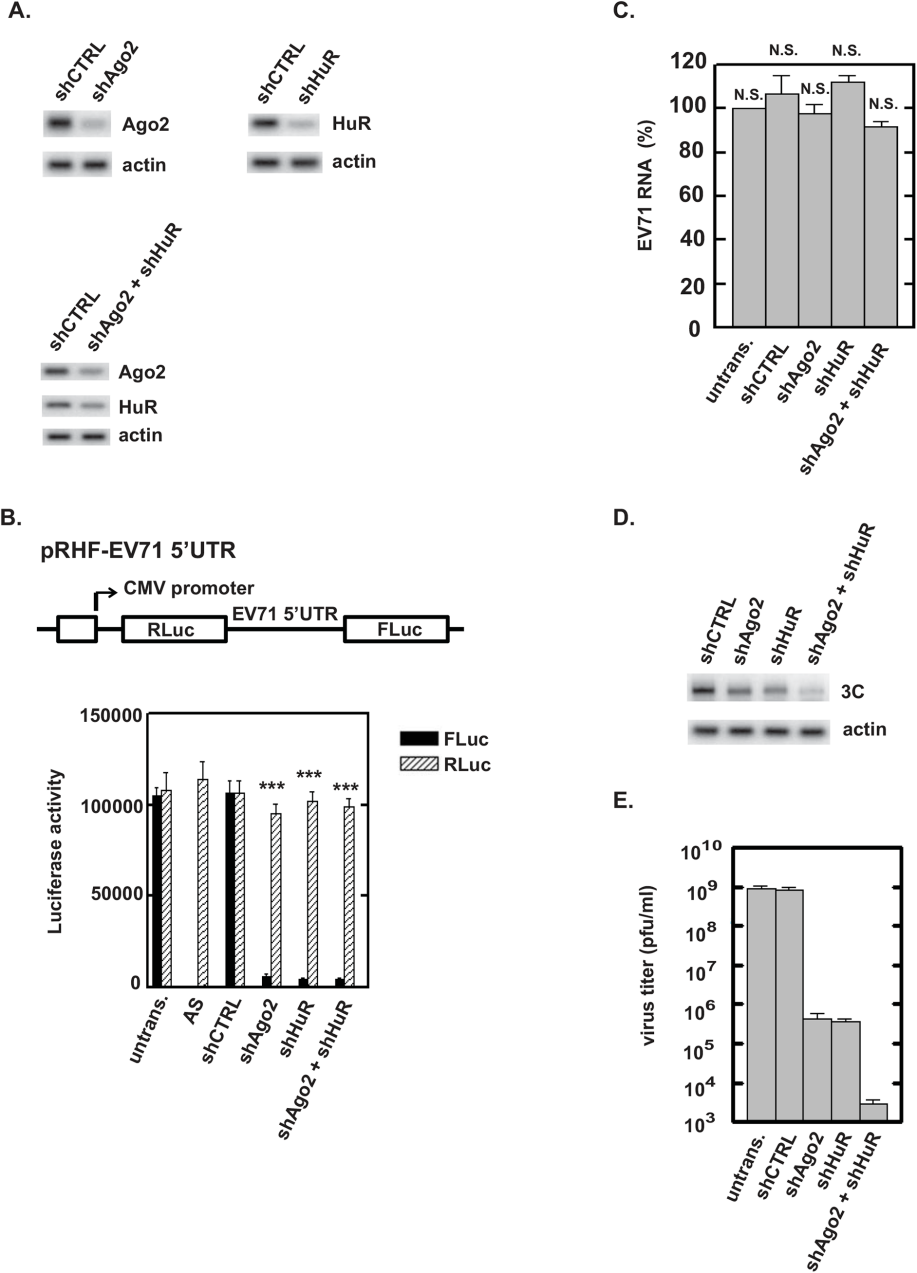
Effects of Ago2 and HuR on EV71 translation and replication. **(A)** SF268 cells were transfected with plasmids expressing shCTRL, shAgo2, shHuR, or shAgo2+shHuR. After two days, extracts were prepared for Western blot analyses of Ago2, HuR and β-actin (loading control). **(B)** Effects of HuR and/or Ago2 knockdown on EV71 IRES activity. The diagram depicts the bicistronic Luc reporter plasmid used to synthesize RNA for transfections. A control plasmid contained the IRES in the antisense (AS) orientation. SF268 cells were transfected with plasmids expressing shCTRL, shAgo2, shHuR, shAgo2+shHuR, or no shRNA (untrans.). Two days after transfections, RLuc-EV71-5′UTR-FLuc RNA was transfected into cells. Luciferase activities were measured two days later. Mean values and standard deviations from three independent experiments are shown in the bar graph. ***, *P* < 0.001. **(C)** Effect of HuR and/or Ago2 knockdown on EV71 RNA levels. Cells were transfected with plasmids expressing shCTRL, shAgo2, shHuR, shAgo2+shHuR, or were left untransfected (untrans.). Two days later, cells were infected with EV71 at an MOI = 2 for 24 h. Total RNA was extracted and viral positive-strand RNA levels were determined by qRT-PCR. Mean values and standard deviations from three independent experiments are shown. N.S., not significant. **(D)** Effect of Ago2 and/or HuR knockdown on EV71 3C protease levels. SF268 cells were transfected with plasmids expressing shCTRL, shAgo2, shHuR, or shAgo2+shHuR. Cells were mock infected or infected with EV71 at an MOI = 2 two days after transfection. Cell lysates were prepared 24 h post-infection (p.i.) and analyzed by Western blotting with anti-3C antibody. Actin served as a loading control. **(E)** Effect of knockdown of Ago2 and/or HuR on EV71 replication. SF268 cells expressing shCTRL, shAgo2, shHuR, shAgo2+shHuR, or were not transfected (untrans.) were infected with EV71 at an MOI = 2 and incubated at 37°C. Medium was harvested 24 h post-infection and assayed for infectious virus by plaque formation with Vero cells. Mean values and standard deviations from three independent experiments are shown.

To determine whether Ago2 and HuR affect EV71 replication, levels of Ago2, HuR, or both proteins together were reduced by transfection of SF268 cells with the respective plasmids expressing shRNAs against Ago2, HuR, or a control shRNA. Western blot analysis indicated that Ago2 and HuR were reduced >80% when compared to serial dilutions of lysate from cells expressing the control shRNA, shCTRL (Fig 3A and data not shown). A bicistronic reporter plasmid, pRHF-EV71-5’UTR, which contains the EV71 5’UTR between the RLuc and FLuc open reading frames, was used as a template for synthesis of RLuc-EV71-5’ UTR-FLuc RNA (Fig 3B). Translation of RLuc is cap-dependent; translation of FLuc is IRES-dependent. Forty-eight hours after transfection of bicistronic RNA, RLuc and FLuc activities were measured by a dual-luciferase assay. Compared to untransfected cells and cells expressing shCTRL, Ago2 or HuR knockdown decreased IRES-dependent translation (i.e., FLuc) to near-background levels, i.e., similar to those observed with the IRES in the nonfunctional, antisense (AS) orientation (Fig 3B, solid bars). Double knockdown of Ago2 and HuR did not appear to reduce IRES-dependent translation beyond that observed upon knockdown of single proteins (Fig 3B, solid bars; compare shAgo2+shHuR graph to shHuR and shAgo2 graphs). A comparison of RLuc activities in control and Ago2-, HuR-, or Ago2+HuR-depleted cells indicated knockdowns had no effect on cap-dependent translation (Fig 3B, hatched bars). These results indicated that Ago2 and HuR are required for IRES activity.

Effects of Ago2 and HuR knockdown on viral RNA synthesis, translation, and titer were examined next. To determine the effect of Ago2 and HuR knockdown on viral RNA synthesis, cells expressing shAgo2, shHuR, or shAgo2+shHuR were infected with EV71 at an MOI = 2 and were cultured with 2 μg/ml actinomycin D. Viral positive-strand RNA levels were examined by real time RT-PCR 24 h post infection. Knockdown of either Ago2 or HuR, or both proteins, had no effect on viral RNA levels (Fig 3C). Western blotting was performed with antibody against EV71 3C protease to examine the effect of Ago2 and HuR depletion on viral protein synthesis. Levels of the viral 3C protein at 24 h post-infection were decreased 25–35% upon knockdown of Ago2 or HuR; the reduction in 3C was 2.5-fold upon double knockdown of Ago2 and HuR (Fig 3D). Thus, the effect of reducing expression of both Ago2 and HuR on 3C abundance was additive. These reductions in 3C levels are consistent with decreased IRES activity. The effects of Ago2 and/or HuR depletion on virus replication were examined as well. Cells expressing shAgo2, shHuR, or shAgo2+shHuR were infected with EV71 at an MOI = 2, and virus titers were determined by plaque assay 24 h post infection. Virus yield from Ago2- or HuR-depleted cells decreased more than 1,000-fold. Upon knockdown of both proteins, virus yield decreased almost a million-fold compared to control cells (Fig 3E). Taken together, these findings indicate that Ago2 and HuR proteins positively regulate EV71 replication and these proteins act additively for maximal virus replication.

### Association of AUF1, Ago2 and HuR with SL-II within the EV71 5’UTR occurs independently of each other

**Fig 4:**
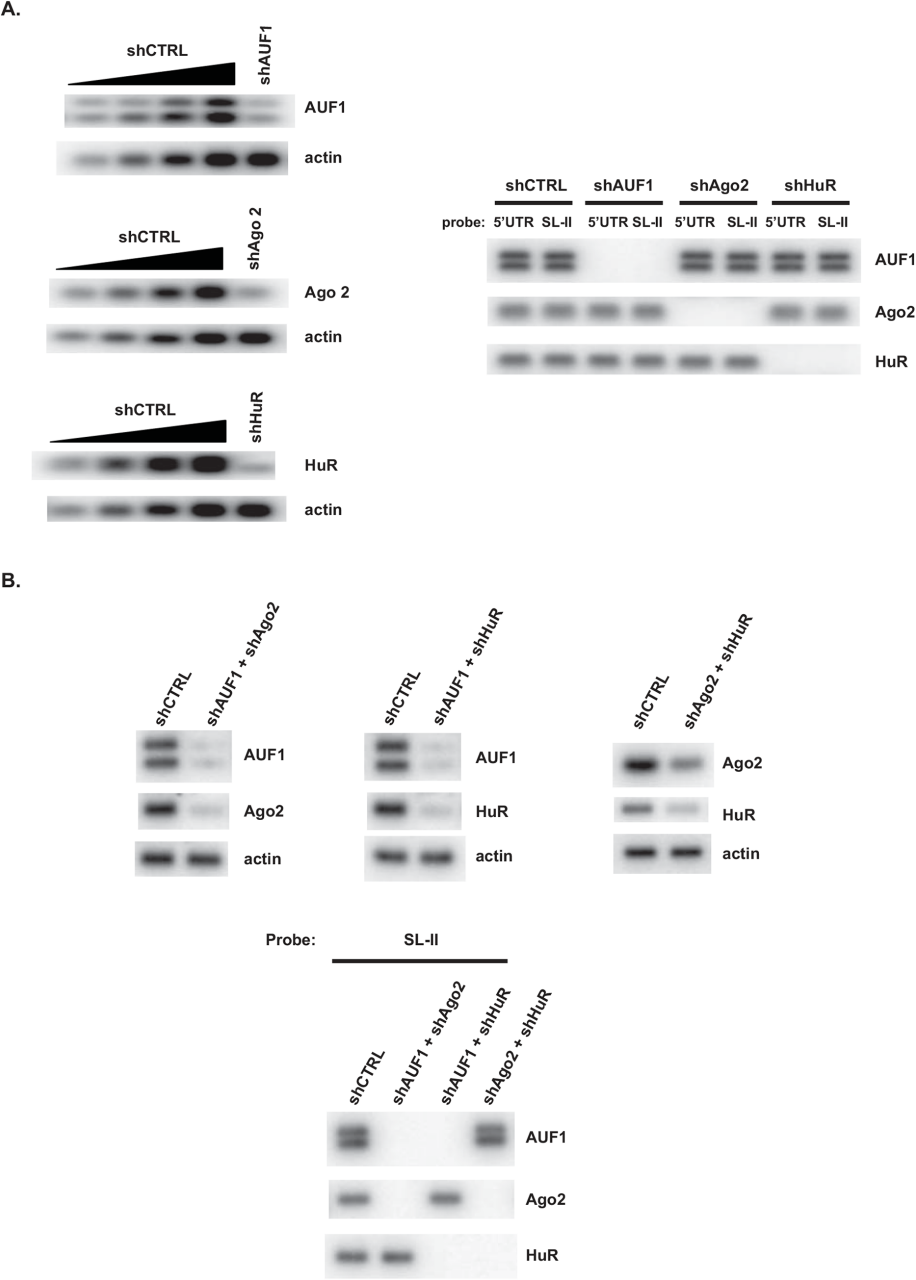
Association of AUF1, Ago2, and HuR with SL-II is independent of each other. **(A)** Left panels: Levels of AUF1, Ago2 and HuR were reduced individually by transfection of SF268 cells with plasmids expressing the indicated shRNA, or shCTRL as a control. Knockdown efficiencies were estimated by comparisons of Western blots of lysates from cells expressing target shRNAs with serial dilutions of lysate from cells expressing shCTRL. Actin served as a loading control. Right panel: The pull-down assay was carried out as described for Fig 2. Recovered proteins were analyzed by Western blot using the antibodies against the indicated proteins (right panel). **(B)** Top panels: Levels of AUF1, Ago2 and HuR were reduced in combinations of two proteins by transfection of SF268 cells with plasmids expressing the indicated shRNAs, or shCTRL as a control. Knockdown efficiencies were estimated by comparisons of Western blots of lysates from cells expressing target shRNAs versus lysate from cells expressing shCTRL. Actin served as a loading control. Bottom panel: Proteins recovered from individual pull-down assays were analyzed by Western blot using the antibodies against the indicated proteins.

Our previous work [15] and the present study show that AUF1, Ago2 and HuR associate with SL-II within the the EV71 5’ UTR. We next asked whether the binding of AUF1, Ago2 and HuR to SL-II is dependent upon one another. A biotin-labeled RNA-protein pull-down assay was performed with lysates of SF268 cells expressing shAUF1, shAgo2, shHuR, or shCTRL to test the effects of silencing expression of one protein upon RNA binding by the other two proteins. Knockdown efficiency was assessed by Western blots comparing serial dilutions of control lysate, as described above (Fig 4A, left panels). Knockdown of one of the three proteins had no discernible effect on RNA binding by the other two proteins to either SL-II or full-length 5’UTR (Fig 4A, right panels). This result suggests that association of any two proteins with the RNA does not require the presence of the third protein.

As such, the effects of knocking down expression of two proteins on RNA binding by the third protein were examined. The Western blots show efficient, dual knockdowns upon transfection of SF268 cells with plasmids expressing two target shRNAs (Fig 4B, top panels). In all cases, however, knockdown of two proteins in any combination had no discernible effect on binding to SL-II RNA by the third protein (Fig 4B, bottom panels). Thus, each protein appears to possess the capacity to bind SL-II without contributions by the others.

### vsRNA1 promotes association of AUF1, Ago2 and HuR with SL-II

**Fig 5:**
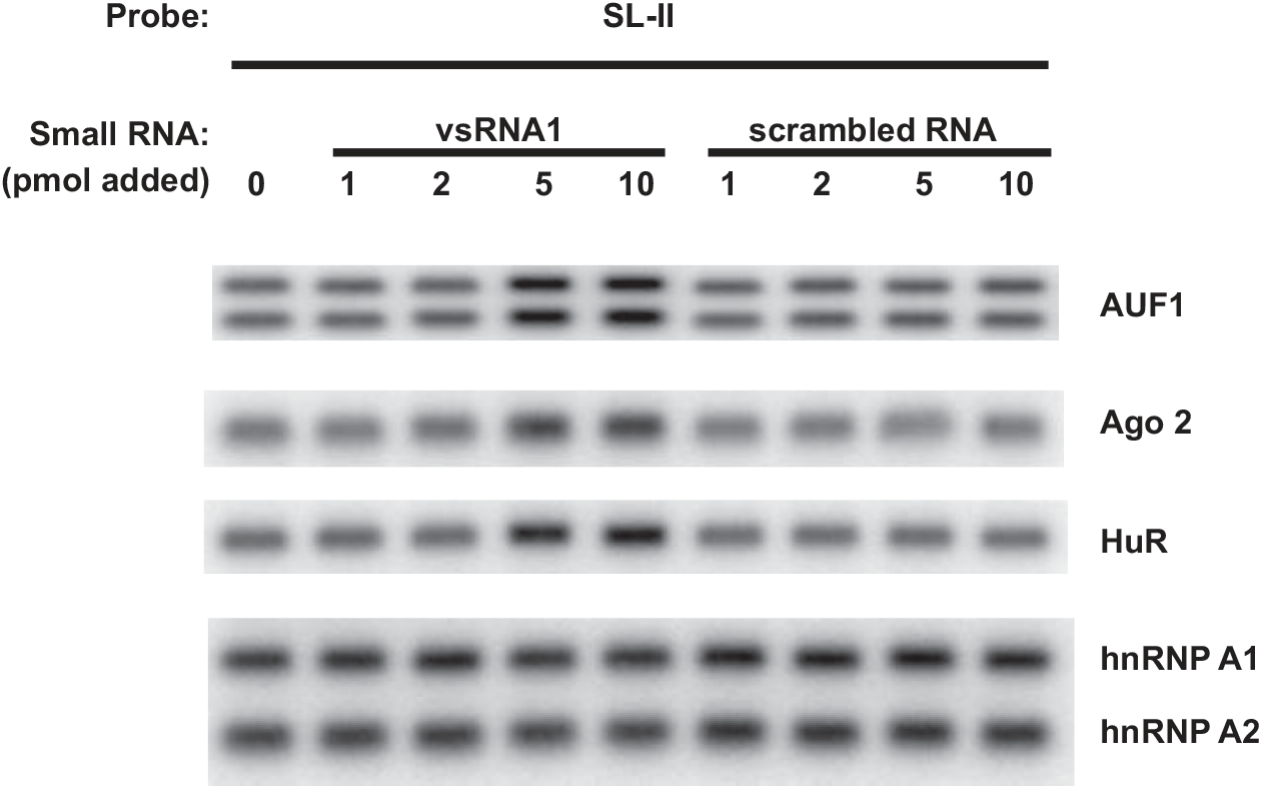
vsRNA1 enhances association of AUF1, Ago2, and HuR with SL-II. The pull-down assay was performed with SF268 lysate and biotin-labeled SL-II as described for Fig 2. Additionally, the indicated pmoles of synthetic mimic vsRNA1 or synthetic scrambled RNA were added to reaction mixtures to examine the effects on AUF1, Ago2, and HuR binding to biotin-labeled SL-II RNA. Eluted proteins were analyzed by Western blot using antibodies to the indicated proteins.

The results so far indicate that Ago2 and HuR bind SL-II; RNA binding by either protein appears essential for IRES-dependent translation and optimal virus replication. Earlier work showed that AUF1 binds SL-II as well, but its binding reduces IRES-dependent translation and virus replication [15]. Earlier work also showed that EV71-infected cells utilize Dicer to generate a number of small RNAs from the EV71 5’UTR [16]. One of these, virus-derived small RNA 1 (vsRNA1), originates from SL-II and targets SL-II to inhibit IRES activity [16]. The mechanism of vsRNA1 action is unknown. One hypothesis is that vsRNA1 might alter the binding of AUF1, Ago2, and/or HuR to SL-II to effect IRES-dependent translation. To determine the impact of vsRNA1 on association of AUF1, Ago2 and HuR with SL-II, the RNA-protein pull-down assay was performed with biotin-labeled SL-II RNA and cell extracts to which increasing amounts of synthetic mimic vsRNA1 or a synthetic control RNA were added (0–10 pmol). Earlier work showed that 2.5 x 10^6^ infected cells produced 1.5 pmol [6 h p.i.], 3 pmol [9 h p.i.], and 5 pmol [12 h p.i.] of vsRNA1 [16]. As shown in Fig 5, addition of 5 pmol of vsRNA1 increased association of AUF1, Ago2, and HuR with SL-II more than twofold (AUF1: 2.2 fold; Ago2: 2.8 fold; HuR: 2.5 fold); addition of 10 pmol of vsRNA1 did not further increase protein binding. Addition of the control scrambled RNA had no apparent effect on binding of AUF1, Ago2 or HuR to SL-II, indicating that the effects are specific to vsRNA1. hnRNP A1 binds SL-II and enhances IRES activity [12]. However, addition of vsRNA1 did not affect binding of hnRNP A1/A2 to SL-II (Fig 5, bottom panel). This suggests that the effects of vsRNA1 on protein binding to SL-II are restricted to specific proteins. However, the significance of increased RNA binding by both positive- and negative-acting ITAFs is currently unknown.

## Discussion

Similar to AUF1 [15], HuR and Ago2 interact with EV71 RNA in cells (Fig 1). In vitro assays revealed these proteins interact specifically with SL-II within the EV71 5’UTR (Fig 2). Optimal IRES-dependent translation and virus replication requires both Ago2 and HuR since knockdown of either protein substantially impaired both processes (Fig 3). Compared to IRES-dependent translation, virus replication is particularly sensitive to the combined knockdown of Ago2 and HuR. Knockdown of either protein alone reduced virus titer about 1,000-fold compared to control cells. However, knockdown of both proteins reduced virus titer almost a million fold. Thus, the effects on virus replication appear greater than the effects on IRES-dependent translation. This might be due to the fact that Ago2 and HuR regulate expression of numerous cellular proteins, and some of these may control host signaling pathways or other proteins that facilitate virus entry or packaging, independently of IRES-dependent translation. Identification of these host proteins awaits further investigation.

Binding of the AUF1, Ago2, and HuR ITAFs to SL-II is independent of one another, since knockdown of either a single protein or a two-protein combination did not affect binding by the remaining protein(s) to SL-II (Fig 4). Infected cells utilize Dicer, the small RNA-processing enzyme, to produce several virus-derived small RNAs (vsRNAs) from the IRES. One of these, SL-II–derived vsRNA1, negatively regulates EV71 IRES activity and virus replication by unknown mechanisms [16]. However, vsRNA1 enhanced association of AUF1, Ago2 and HuR with SL-II in biotin-RNA pull-down reactions supplemented with synthetic, mimic vsRNA1, but not a control scrambled RNA (Fig 5).

RNA-induced silencing mediated by RISC is an important defense against viral infections [25–28]. Our recent study [16] found that Ago2 associates with vsRNA1 in infected cells and that vsRNA1 reduces both IRES-dependent translation and virus replication. However, results described in the current work showed that knockdown of Ago2 significantly reduced IRES activity, suggesting that vsRNA1 and Ago2 may act on IRES activity antagonistically. In vitro, vsRNA1 enhanced association of Ago2 with SL-II. This would be predicted to increase IRES activity, based on the observation that silencing Ago2 expression decreased IRES activity (Fig 3). As vsRNA1 also enhanced the association of both AUF1 (a negative ITAF) and HuR (a positive ITAF) with SL-II, it is likely that integration of a myriad of inputs, provided by vsRNA1, AUF1, Ago2, HuR, and other RNA-binding proteins, determines IRES activity. Future work will be required to fully dissect the molecular details of IRES activity.

In addition to stabilizing subsets of cellular mRNAs to which it binds, HuR affects virus gene expression as well. For example, it binds to the HCV 3’UTR, though the significance of this binding is not clear [29]. During HIV infection, HuR interacts with the viral reverse transcriptase and negatively regulates HIV IRES-mediated translation; however, HuR activates HCV IRES-dependent translation [30,31]. HuR associates with the 3’UTR of late HPV transcripts and regulates late gene expression [32]. Moreover, HuR binds to the 3’UTR of Sindbis virus to stabilize its transcript during infection of mammalian and mosquito cells [33]. The association of HuR with the Sindbis virus 3’UTR results in sequestration of HuR and leads to destabilization of cellular mRNAs that it would normally bind. Sequestration of HuR by the 3’UTR of Sindbis virus transcripts also leads to significant changes in cellular polyadenylation and splicing [34]. The impact of EV71 on posttranscriptional regulation of cellular gene expression is less clear. However, EV71 infection does cause relocalization of AUF1 from the nucleus to the cytoplasm [15]. The effects of this relocalization on host gene expression and the impact on virus replication and pathogenesis await further investigation.

AUF1, HuR, and Ago2 normally control host mRNA degradation and/or translation. Interestingly these three ITAFs associate with SL-II and vsRNA1 promotes their association with SL-II. The molecular details underlying the interactions between these ITAFs and vsRNA1 are not known. It is tempting to speculate that vsRNA1 may act to remodel local RNA structure within SL-II to favor or disfavor RNA binding among AUF1, Ago2, and HuR. Alternatively, vsRNA1 may recruit other host factors to SL-II to affect translation. For example, a miR-122–Ago2 complex binds the 5’UTR of HCV viral RNA to enhance RNA stability and translation [35–37]. In any event, further studies will be required to dissect the mechanisms by which vsRNAs and host RNA-binding proteins regulate IRES activity and virus replication.
